# Commentary: Phase-amplitude coupling at the organism level: The amplitude of spontaneous alpha rhythm fluctuations varies with the phase of the infra-slow gastric basal rhythm

**DOI:** 10.3389/fnins.2017.00102

**Published:** 2017-03-02

**Authors:** Jan D. Huizinga

**Affiliations:** Department of Medicine, Farncombe Family Digestive Health Research Institute, McMaster UniversityHamilton, ON, Canada

**Keywords:** interoception, interstitial cells of Cajal, vagal afferents, pacemaking, brain gut axis, motility, gastric motility

The study by Richter et al. (2017) found significant electrophysiological communication between gastric pacemaker activity and the alpha rhythm within certain regions of the cerebral cortex including the right anterior insula. In other words, they found a role for interstitial cells of Cajal in interoception, the sensory system responsible for detecting internal regulation responses. The communication between ICC and the insular cortex occurred through phase amplitude coupling, the phase of the lower frequency gastric pacemaker modulated the amplitude of alpha waves in the anterior insula. Although phase amplitude coupling has almost exclusively been shown to occur within the brain, this study discovered that it also facilitates gut—brain communication. Interestingly, phase amplitude coupling was also observed within the gut; the amplitude of the higher frequency dominant intestinal pacemaker [the “slow wave” generated by interstitial cells of Cajal associated with the myenteric plexus (ICC-MP; Huizinga et al., 1995; Thomsen et al., 1998)] was seen to be modulated by the phase of a lower frequency induced rhythmic depolarization likely originating in the network of ICC associated with the deep muscular plexus (ICC-DMP) (Huizinga et al., 2014). This interaction changed propulsive activity into the classical segmentation pattern of the intestine associated with absorption of nutrients. The study from Richter et al. (2017) is highly significant since it demonstrates the ongoing monitoring of gastric pacemaker activity by the right anterior insula. In the stomach, the three-cycles/min pacemaker activity responsible for the orchestration of its dominant peristaltic activity, is generated by a network of ICC-MP and ICC-IM (the intramuscular ICC) (Edwards and Hirst, 2006). The communication between gastric ICC and the brain involves the intramuscular array (IMA)-ICC-complexes which incorporate ICC-IM, nerve endings of the vagal afferents and varicosities of motor neurons (Powley et al., 2008, 2016), ideally suited for bi-directional communication with the brain (Figure 1). The vagal afferents connect to the insular cortex via the nucleus tractus solitarius (Shipley, 1982) and the insular cortex innervates the dorsal motor nucleus, which, in turn, provides innervation to enteric nerves of the stomach (Berthoud et al., 1990). The insular cortex was activated during gastric balloon distention and deactivated during ingestion of a meal, indicating a detailed monitoring of stomach conditions (Geeraerts et al., 2011). Vagal afferents are all connected to ICC (Powley et al., 2016) indicating that the vagus will not monitor individual ICC but the features of the interconnected network of ICC (Huizinga et al., 2015; Pawelka and Huizinga, 2015; Wei et al., 2016). How detailed information from this network is conveyed to the brain should be a topic for future research. This information will contain signals from several slow waves propagating over the stomach at the same time. This information will contain changes in ICC network properties that occur in response to a meal (Chen and McCallum, 1992) (Berthoud, 2008), which may relate to satiety (Andrews and Sanger, 2002). There is the potential that detailed information regarding meal quality and/or quantity, or even types of nutrients ingested could be signaled via alterations in ICC signaling. Detailed information is now emerging how ICC network properties change in patients with gastroparesis (O'Grady et al., 2012; O'Grady and Abell, 2015) and diabetes (He et al., 2001). Dyspepsia may relate to abnormal vago-vagal reflexes, including efferent innervation and abnormal signaling from the stomach to the insula or abnormal processing of such signals (Page and Blackshaw, 2009; Lee et al., 2016). Abnormalities in initiation and conduction were observed in patients with gastroparesis in the presence of a normal 3 cpm frequency (O'Grady et al., 2012) suggesting that in certain conditions, the recorded ICC pacemaker frequency may be normal but that it is the injury to the ICC network that is related to gastroparesis, which may relate to delayed gastric emptying and/or the initiation of nausea and vomiting through vagal afferents. Gastric dysmotilities are also related to depression indicating the myriad of ways that different regions of the brain can influence each other (Ruhland et al., 2008). Gastric slow wave activity changes markedly in response to neurotransmitters and hormones (El-Sharkawy and Szurszewski, 1978; El-Sharkawy et al., 1978), hence it is likely that the insular cortex monitors such changes. Vagal stretch and tension receptors are always incorporated in mechanistic explanations as to how the stomach signals to the brain (Young et al., 2008; Kentish et al., 2013). The study of Richter et al. (2017) suggests that the ICC-IMA complexes are a sensorimotor unit and that sensations might be primarily integrated by and expressed by ICC activity and as such monitored by the insular cortex, where, in conjunction with other regions of the brain, conscious and subconscious decisions are made how to react to these stimuli. Monitoring of slow wave activity, even at rest (Richter et al., 2017), ensures an exquisitely sensitive system that is instantly available to inform the brain of any activity, change in activity or abnormal conditions. Further investigations into the role of ICC will be essential to unravel this gut brain communication pathway, including the sensitivity of ICC to inflammation, the remarkable ability of ICC to recover from severe injury and loss (Wang et al., 2002; Bettolli et al., 2012) and the molecular basis of ICC network regeneration (Hayashi et al., 2013).

**Figure 1:**
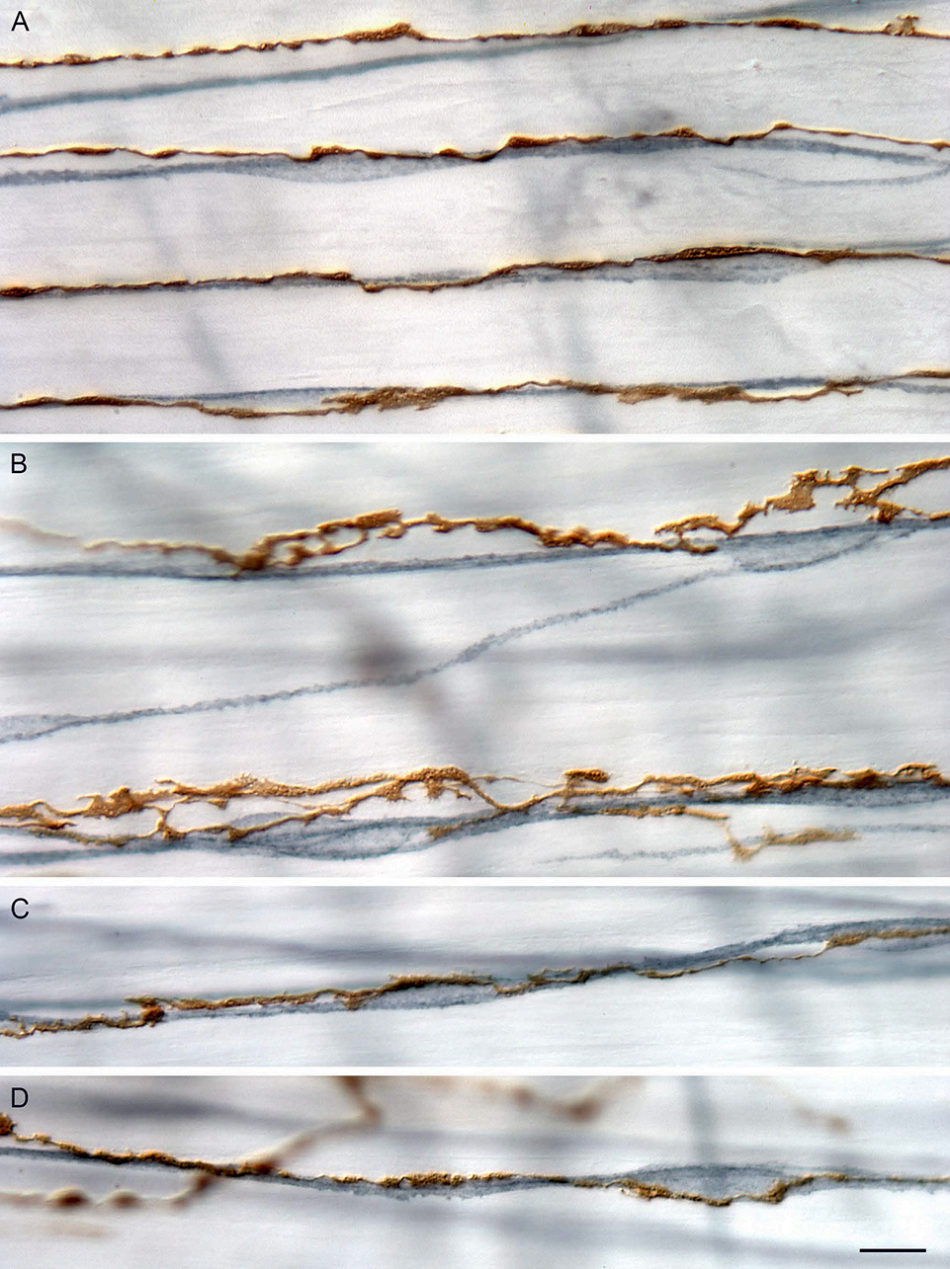
**Branches of IMAs (brown, 3,3′-diaminobenzadine stained) run in tight apposition with ICC-IM (blue-gray, c-Kit immuno-labeled with peroxidase)**. Branches of IMA arrays vary in their degree of varicosity and in the tightness of apposition. **(A)** Four neighboring principal branches of an IMA array coursing in tight apposition with a network of neighboring ICC-IM intercalated among smooth muscle bundles (unstained). In this example, the IMA branches express modest swellings or varicosities, most of which are in close proximity to the somata and processes of ICC-IM. **(B)** Two neighboring principal branches of an IMA array course near to, and appear to contact intermittently, the local ICC-IM network. In contrast to the array branches shown in **(A)**, those shown in **(B)** are more lamelliform, the apparent contacts with the ICC-IM are more intermittent, and many of the IMA lamellae appear to lie on the smooth muscle bundles (unstained) adjacent to the ICC-IM network. **(C,D)** Two examples of principal IMA branches that course in tight conjunction with ICC-IM and form swellings or varicosities on both ICC-IM somata and fibers. Scale bar = 10 μm. Reproduced with permission from Powley et al. (2016).

## Author contributions

The author confirms being the sole contributor of this work and approved it for publication.

### Conflict of interest statement

The author declares that the research was conducted in the absence of any commercial or financial relationships that could be construed as a potential conflict of interest.
